# A Novel Method for Fast Change-Point Detection on Simulated Time Series and Electrocardiogram Data

**DOI:** 10.1371/journal.pone.0093365

**Published:** 2014-04-01

**Authors:** Jin-Peng Qi, Qing Zhang, Ying Zhu, Jie Qi

**Affiliations:** 1 College of Information Science & Technology, Donghua University, Shanghai, P.R. China; 2 The Australia e-Health Research Centre, CSIRO, Brisbane, QLD, Australia; 3 Hunter New England Health, Royal North Shore Hospital, New South Wales, Australia; Universiteit Gent, Belgium

## Abstract

Although Kolmogorov-Smirnov (KS) statistic is a widely used method, some weaknesses exist in investigating abrupt Change Point (CP) problems, e.g. it is time-consuming and invalid sometimes. To detect abrupt change from time series fast, a novel method is proposed based on Haar Wavelet (HW) and KS statistic (HWKS). First, the two Binary Search Trees (BSTs), termed TcA and TcD, are constructed by multi-level HW from a diagnosed time series; the framework of HWKS method is implemented by introducing a modified KS statistic and two search rules based on the two BSTs; and then fast CP detection is implemented by two HWKS-based algorithms. Second, the performance of HWKS is evaluated by simulated time series dataset. The simulations show that HWKS is faster, more sensitive and efficient than KS, HW, and T methods. Last, HWKS is applied to analyze the electrocardiogram (ECG) time series, the experiment results show that the proposed method can find abrupt change from ECG segment with maximal data fluctuation more quickly and efficiently, and it is very helpful to inspect and diagnose the different state of health from a patient's ECG signal.

## Introduction

Detecting abrupt change from time series, called CP detection, has attracted considerable attention in the fields of data mining and statistics. CP detection [1], [2], [3], [4], [5] has been widely studied in many real-world problems, such as atmospheric and financial analysis [1], intrusion detection in computer networks [2], signal segmentation in data stream [3], as well as fault detection in engineering systems [4], [5]. A good method of CP detection is by comparing probability distributions of time series samples over past and present intervals [6], [7], in which a typical strategy is to trigger an alarm for a CP as two distributions are becoming significantly different. Various methods of change detection follow this statistical framework, including the CUSUM (cumulative sum) [7], the GLR (generalized likelihood ratio) [8], [9] and the change finder [4]. Generally, these approaches are limited by relying on pre-specified parametric models such as probability density models, autoregressive models, and state-space models. Therefore, these methods tend to be less flexible in real-world CP detection problems.

In community of statistics, some non-parametric approaches for CP detection have been explored, in which non-parametric density estimation is used for calculating the likelihood ratio [10], [11]. However, this kind of estimation is a hard problem [12], [13], and may not be promising in practice. As a nonparametric method, the KS statistic quantifies a distance between the empirical distribution function of the sample and the cumulative distribution function of the reference distribution, or between the empirical distribution functions of two samples [14], [15]. Moreover, the KS test is for the equality of continuous, one-dimensional probability distributions that can be used to compare a sample with a reference probability distribution, or to compare two samples. The null distribution of this statistic is calculated under the null hypothesis that the samples are drawn from the same distribution or that the sample is drawn from the reference distribution. The two-sample KS test is one of the most useful and general nonparametric methods for comparing two samples, as it is sensitive to differences in both location and shape of the empirical cumulative distribution functions of the two samples. Recently, non-parametric KS statistic and its modified version are broadly investigated on several of application fields. For example, the use of the KS statistic for testing hypotheses regarding activation in blood oxygenation level-dependent functional MRI data [16]; modeling the cumulative distribution function of rub-induced AE signals and quantifying the goodness of fit with the KS statistic, to offer a suitable signal feature for diagnosis [17]; abrupt change point (CP) detection from electroencephalography signal (EEG) [18], and gene expression time series database [19].

On the other hand, Wavelet Transform (WT) is another promising approach for CP detection. In the past decade, WT approach has emerged as an important mathematical tool for analyzing time series [20], [21], [22], [23], [24]. It has found applications in anomaly detection, time series prediction, image processing, and noise reduction [20], [24], [25], [26]. In particular, wavelets can represent general functions at different scales and positions in a versatile and sophisticated manner, so the data distribution features can be easily extracted from different time or space scales [26], [27]. The heart of wavelet analysis is Multi-Resolution Analysis (MRA), by which a signal can be decomposed into sub-signals of different size resolution levels [7], [27], [28]. The properties of wavelets such as localization, orthogonality, multi-rate filtering, are essential for analysis of non-stationary and transient signals. WT can represent a general function in terms of simple, fixed building blocks at different scales and positions. These building blocks are generated from a single fixed function called mother wavelet by translation and dilation operations [29], [30]. In addition, Haar Wavelet (HW), as a simpler WT, owns some attracting features including fast for implementation and ability to analyze the local feature. HW is a very useful to find discontinuities and high frequency changes in time series, and a potential candidate in modern electrical and computer engineering applications, such as signal and image compression, as well as abnormality detection from time series [7], [28].

However, most of these methods above are time-consuming, and not scalable to large-scale datasets due to their time complexity. Moreover, some of them, e.g., KS, is occasionally insensitive even invalid for less significant data fluctuation, especially when abrupt change occurs near two endpoint areas. To detect abrupt change from time series quickly and efficiently, a novel non-parametric method is proposed based on multi-level HW and a modified KS statistic. In this method, we combine superiorities of both KS statistic and HW methods, and try to find an abrupt change in terms of maximal data fluctuation existing between two adjacent segments of a diagnosed time series. This paper is organized as follows. Section II implements the integrated HWKS method in detail. First, the two BSTs termed TcA and TcD, are constructed by means of multi-level HW from a diagnosed time series. Then, the framework of HWKS method is implemented by introducing a modified KS statistic and two search criteria based on TcA and TcD. Last, two HWKS-based algorithms are designed to implement CP detection from the diagnosed time series. Section III evaluates the performance of HWKS by comparing KS, HW, and T methods, via simulated time series and real ECG datasets. Section IV gives conclusion from previous sections.

## Method

**Figure 1:**
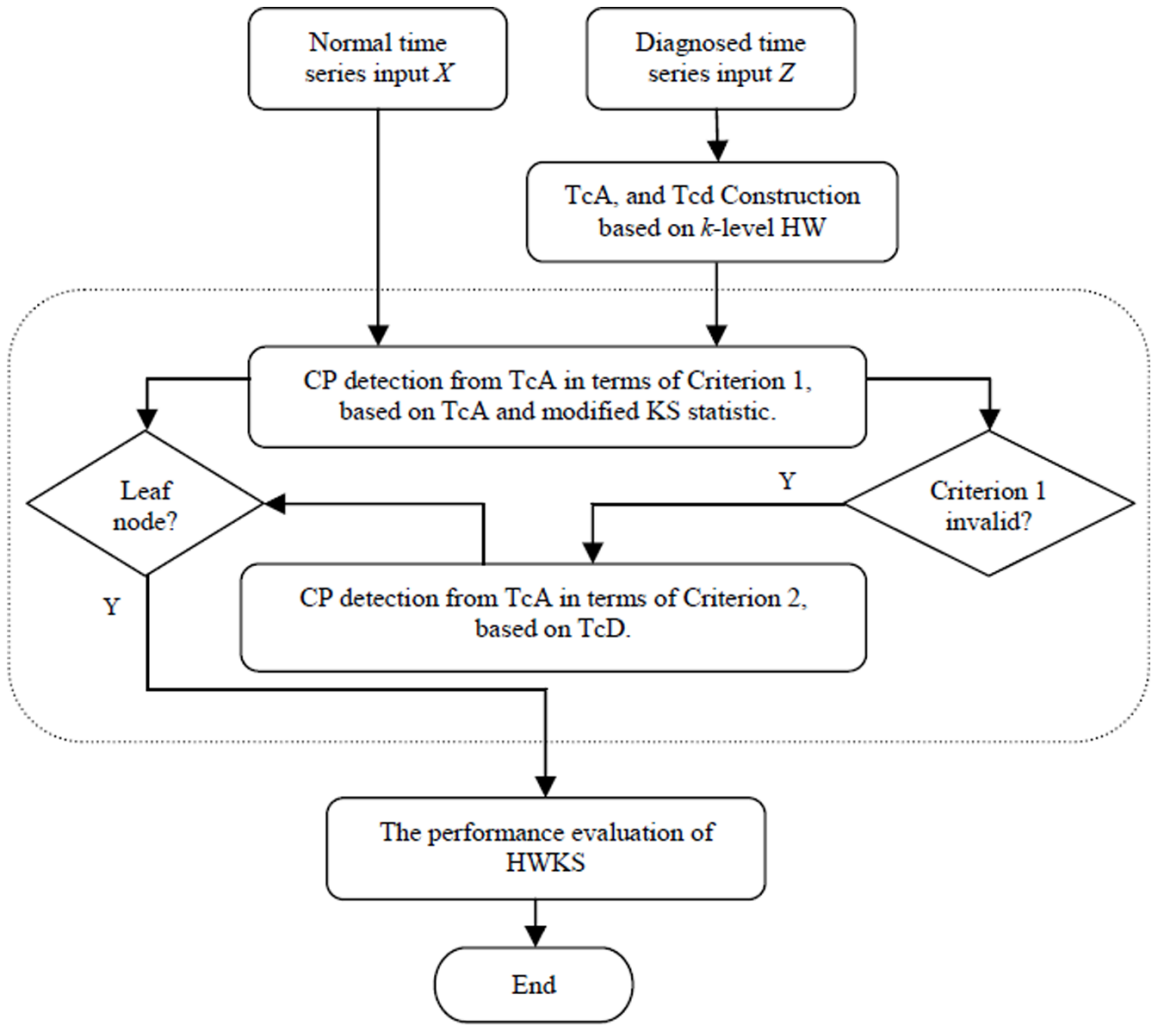
The integrated scheme of HWKS method for fast CP detection, which includes three parts: construction of two BSTs, namely TcA and TcD; CP detection of HWKS in terms of two search criteria; and evaluation of HWKS method.

The flow diagram of the integrated HWKS framework (Fig. 1) is composed of three parts. First, the two BSTs, TcA and TcD are constructed from a diagnosed time series. Second, abrupt CP is detected from root to leaf nodes of TcA in terms of a modified KS statistic and two search rules. Last, the performance of HWKS is evaluated by comparing with KS, HW, and T methods.

### A. Construction of TcA and TcD

**Figure 2:**
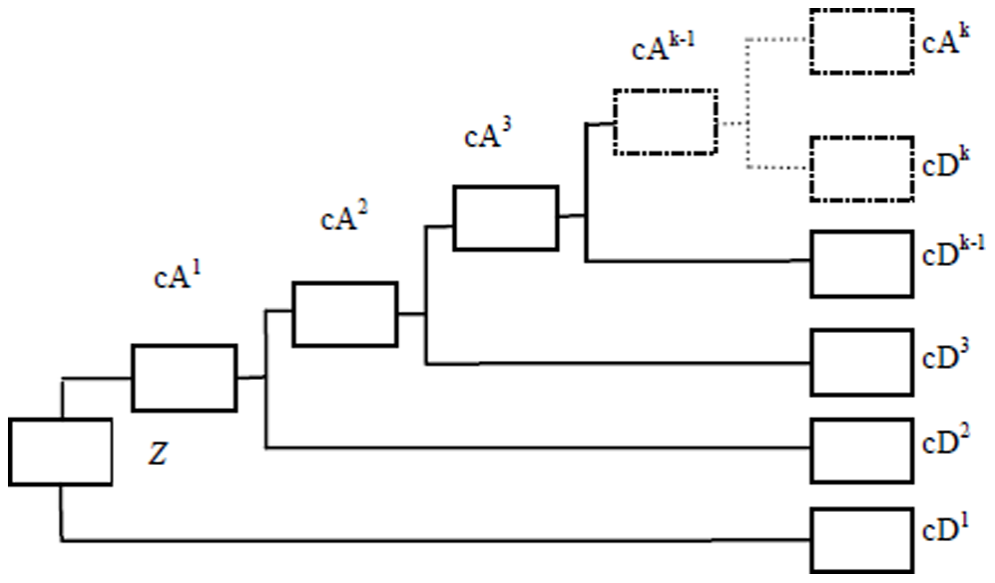
The diagram of multi-level HW for time-series signal *Z*, it is composed of *k*-level cA and cD vectors, i.e., the average and difference coefficients vectors.

Like all wavelet transforms, multi-level HW decomposes a discrete signal into two sub-signals with half its length. One sub-signal is a running average or trend; the other sub-signal is a running difference or fluctuation. HW is performed in several stages or levels [28]. It can be described using scalar products with scaling signals and wavelets. The discrete signals are synthesized by beginning with a very low-resolution signal, successively adding on details to create higher resolution versions, and ending with a complete synthesis of the signal at the finest resolution. Generally, by using *k*-level HW, a discrete time series signal Z = {z_1_, z_2_,…, z*_N_*}, can be decomposed into the *k* ^th^-level trend cA*^k^*, and *k* level fluctuations, i.e.,**c**D^1^, cD^2^,…, cD*^k^*, *k = *1, 2, .., 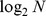. As shown in Fig. 2, the *k*-level HW is the mapping *H*_k_ defined by [8],
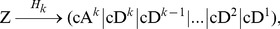
(1)


The multi-resolution analysis (MRA) is the heart of waveletanalysis [23], [29], in terms of MRA, we can conceptualize the process of HW as a projection of time series with size*N* to total *N* different vectors *v*_i_ and *w*_i_, termed as scalingsignals and wavelet basis vectors, respectively. Thediscrete signal Z, average and detail signals are expressible as:
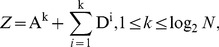
(2)

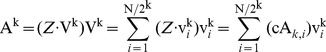
(3)

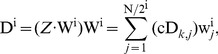
(4)Thereafter, the following equations can be obtained,
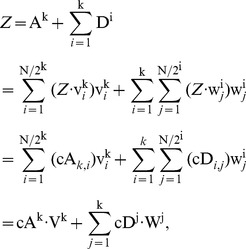
(5)

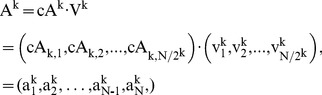
(6)

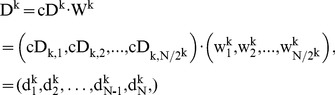
(7)where 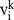 is *k*-level Haar scaling signals, 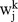 is *k*-level Haar wavelets, |
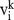
| = |
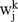
| = *N*.

**Figure 3:**
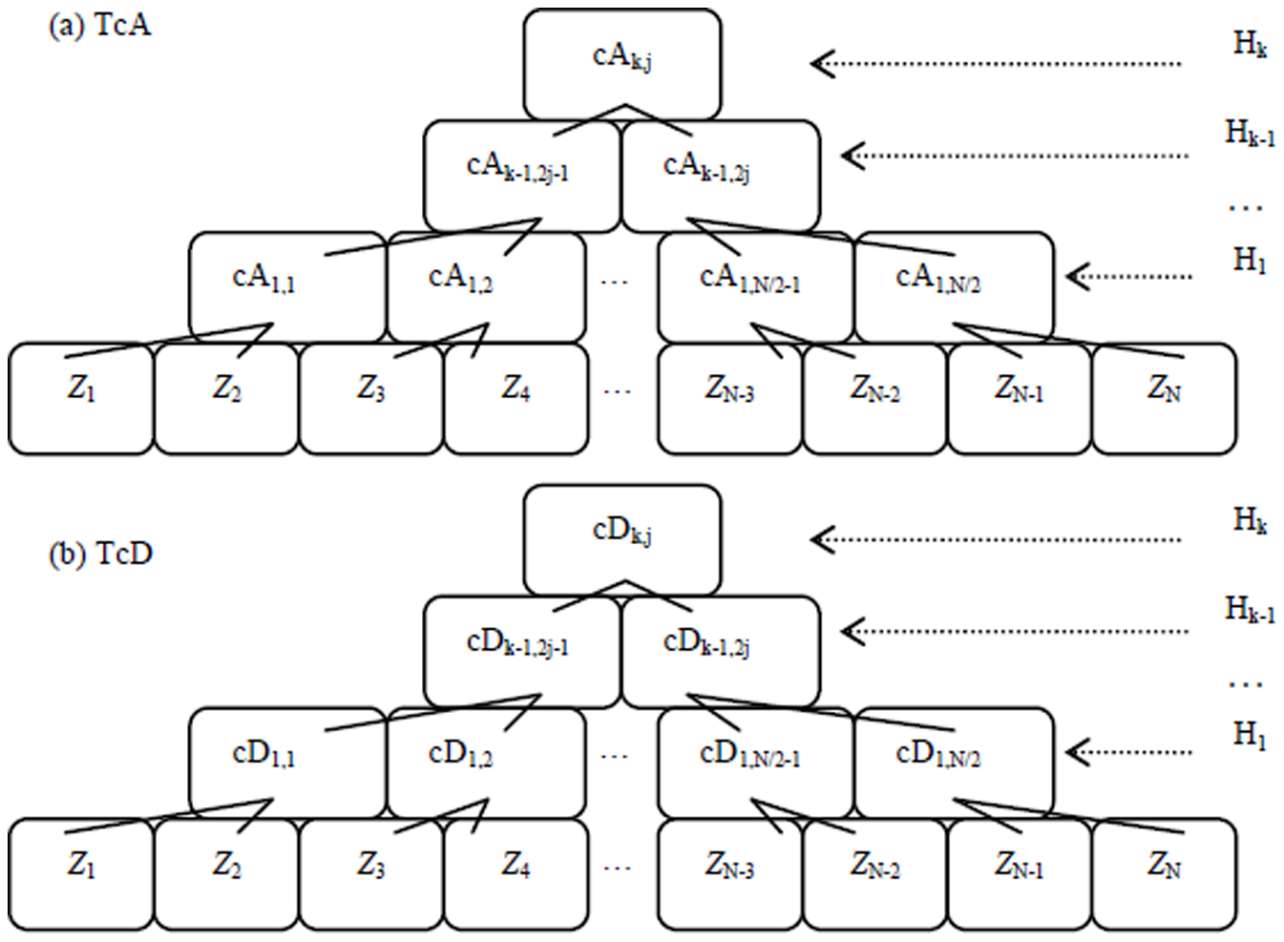
The diagrams of TcA and TcD, derived from a diagnosed time-series *Z* by means of *k*-level HW.

In addition, we can represent HW with *k*-level approximation and detail coefficient vectors by the following matrices, namely McA and McD:
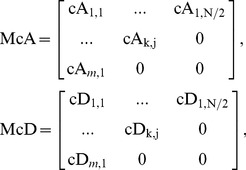
(8)where 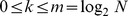, 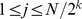. Suppose the size of a diagnosed sample Z is divisible k times by 2, we can further denote the *j* ^th^ element in cA^k^ and corresponding averaged signal in A^k^, as well as the *j* ^th^ element in cD^k^ and corresponding detail signal in D^k^ as follows:
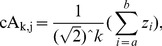
(9)

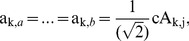
(10)

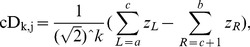
(11)

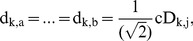
(12)where 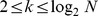, and 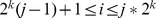; 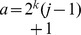, 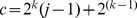, and 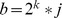. Therefore, a diagnosed Z can be decomposed into cA and cD matrices by means of *k*-level HW. Thereafter, as shown in Fig. 3, TcA and TcD are built in terms of McA and McD, as well as original elements in Z = {*z*_1_, *z*_2_,…, *z*_N_}. In TcA, and TcD, non-leaf nodes in different level are constructed from McA, and McD, respectively; and then leaf nodes are derived directly from the elements in Z. The constructions of TcA and TcD are implemented by Algorithm 1 in detail.

**Algorithm 1.**

**Input:** Z = {z_i_ : 1 ≤ *i* ≤ *N*},a diagnosed time series

**Output:** two BSTs, TcA and TcD

Initiate the level of HW, *k*, (1 ≤ *k* ≤ log2(*N*));

Declare two matrices, McA and McD;

For *i* = 1 to *k* do

 [cA_i_, cD_i_] = Call HW(*Z*, *i*);

 McA(*i*) = cA_i_; McD(*i*) = cD_i_;

end

Construct TcA and TcD from McA and McD, as well as Z.

Output TcA and TcD

### B. HWKS method based on a modified KS statistic

KS test is one of the most useful and general non-parametric methods for comparing two samples, because it is sensitive to those differences in both location and shape of the empirical cumulative distribution functions (e.c.d.f) of two samples [31], [32]. Suppose 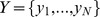 is a time series, we observe,
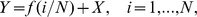
where 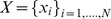 are discrete and centred i.i.d. random variables, and 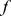 is a noisy signal with unknown distribution. Thereafter, we can deal *X* as a normal time series with distribution function 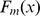, and *Y* as an abnormal time series with distribution function 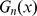. Then, we can assemble a diagnosed time series *Z*, and define it as below:
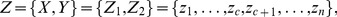
(13)To detect an abrupt CP from Z, a modified KS statistic is defined to evaluate the distribution distance between *X* and Z [15], [33], [34]:
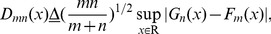
(14)if a change point *c* occurs in Z, there exists a value *z*_c_ satisfies 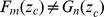, and 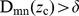, *z*_c_ 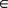
[z_1_, z_n_], 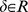.

As hypothesized 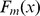 and 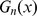 are not available, but instead, the e.c.d.f of 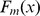 and 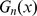 can be derived from two time series *X* and Z. Then, 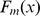 and 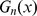 are defined by,
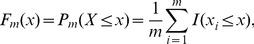
(15)

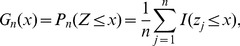
(16)where 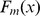 and 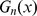 count the proportion of the sample points below level *x*. For any fixed point 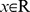, the law of large numbers implies that
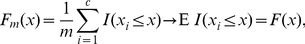
(17)

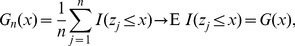
(18)where 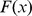 and 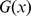 are true underlying distribution of two time series *X* and *Z*, i.e. the proportion of the sample in the set 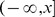 approximates the probability of this set. It is easy to say that this approximation holds uniformly over all 
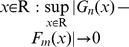
, *i.e.*, the largest difference between 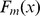 and 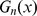 goes to 0 in probability. The key observation in KS test is that the distribution of this supremum does not depend on the ‘unknown’ distribution *P* in diagnosed Z, if *P* is continuous distribution. In addition, we have,
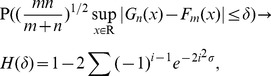
(19)where 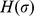 is the c.d.f. of KS distribution [35].

In terms of the definition of non-leaf nodes in TcA in equations (9), we can denote the *j* ^th^ element 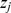 of Z and define a new element 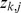 used in HWKS method as,
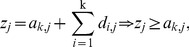
(20)

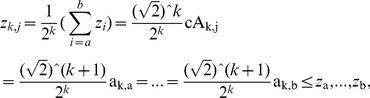
(21)where 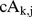 is a non-leaf node of TcA, 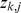 is a new element defined in terms of 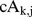, and 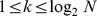, 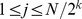; 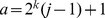, and 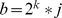. Then, a revised KS statistic for HWKS is defined as:
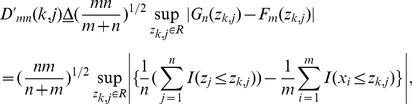
(22)where 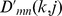 measures the distribution distance between *X* and *Z* at a selected node 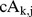 in TcA, and larger value of 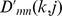 means that more significant change occurs in Z.

Thereafter, we define a KS test for *X* and Z as,
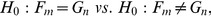
(23)if null hypothesis is true then, the distribution of 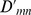 can be tabulated as it depends only on *n*. Moreover, if *n* is large enough then the distribution of 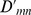 is approximated by KS distribution. On the other hand, suppose 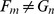, since 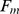 and 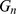 are the true c.d.f. of *X* and Z, bylaw of large numbers the e.c.d.f, 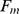 will converge to 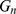, and for large *n* we will have,
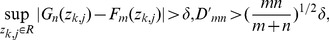
(24)If 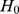 fails then,
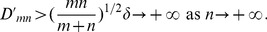
(25)Therefore, to test 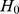 we make a detection rule,
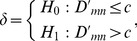
(26)where *c* depends on the level of significance 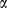, and can be found by using KS distribution when *n* is large.
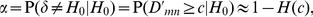
(27)If 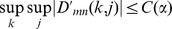, *H*_0_ is true i.e., no change point occurs. On the other hand, if 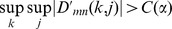, then hypothesis *H*_1_ is true *i.e*., an abrupt change is detected.

### C. HWKS-based CP Detection

**Figure 4:**
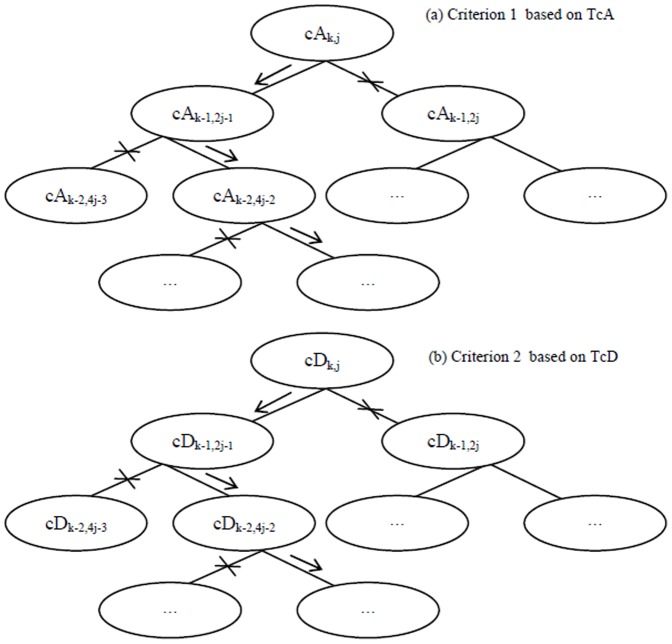
The scheme of two search criteria for CP detection of HWKS. (a) Criterion1 based on TcA, and (b) Criterion 2 based on TcD ensure that an optimal path of abrupt CP can be detected from root to leaf nodes in TcA.

To detect abrupt change from diagnosed Z, an optimal path needs to be obtained from root to leaf nodes in TcA accurately and quickly. Therefore, as shown in Fig. 4, two search criteria are introduced in terms of TcA and TcD. The first search criterion is defined based on TcA as follows:

#### Criterion 1

Suppose the current non-leaf node in TcA we selected is 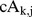, and its left-child and right-child nodes are 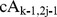 and 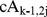, respectively,

- if 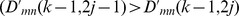 and 
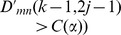
 hold true, then the left-child node 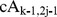 is selected to be involved into the current search path in TcA;
- if 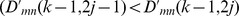 and 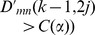 hold true, then the right-child node 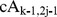 is selected to be involved into the current search path in TcA.

#### Proof

**Figure 5:**
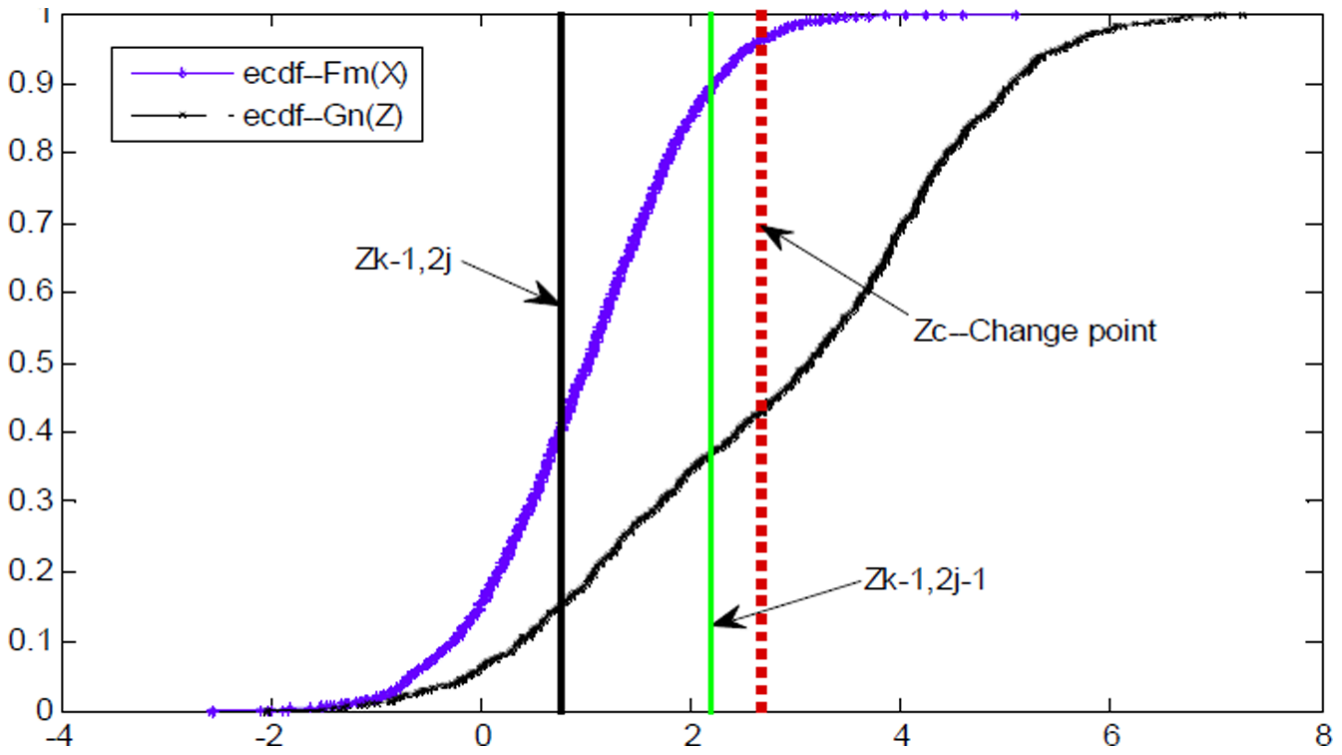
The scheme of search strategy in Criterion 1 in terms of distribution distance between *X* and *Z*. The dotted red line refers to a supposed CP, namely Z_c_, the solid black line, and green one stands for the points of z_k-1,2j_, and z_k-1,2j-1_, respectively.

In terms of the definitions of 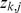 and 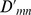 in equation (21) and (22), 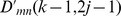, and 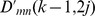 can be written as
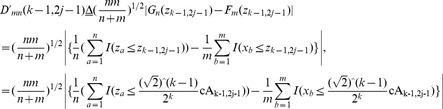
(28)

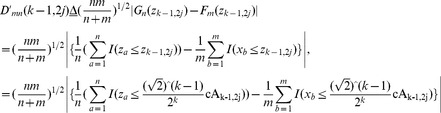
(29)where 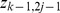 and 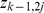 are two diagnosed points in accordance with cA_k-1,2j-1_ and cA_k-1,2j_ in TcA. In terms of Criterion 1, if 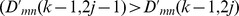 holds true, as plotted in Fig. 5, it indicates that more significant distribution distance exists in the left sub-tree covered by 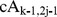, than in the right one covered by 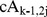, and vice versa. That is, abrupt CP occurs in the left segment of Z with more probability than in the right one. On the other hand, If 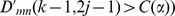 is satisfied, it means that the distribution distance overtakes a critical value given in an identical data distribution.

Criterion 1 guarantees that if abrupt CP occurs in Z, the left or the right sub-tree with bigger distribution distance is selected to be involved into the current search path, and then the other half part is discarded. Thereafter, an optimal search path can be detected from root to leaf nodes in TcA after about *log*_2_ (*n*) search steps. Unfortunately, if 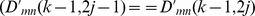 or 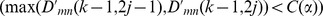 are true, then Criterion 1 is invalid for CP detection. Therefore, another search criterion is introduced based on TcD, and defined as below.

#### Criterion 2

Suppose 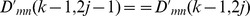 or 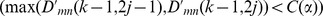 is satisfied, the non-leaf node 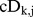 in TcD is selected, in accordance with the current non-leaf node 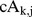 in TcA, with its left-child node 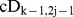 and right-child node 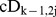, respectively,

- if 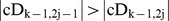 holds true, then the left-child node 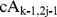 is selected to be involved into the current search path in TcA;
- if 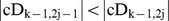 holds true, then the right-child node 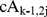 is selected to be involved into the current search path in TcA.

#### Proof

In accordance with the definition of 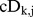 in equation (11), 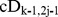 and 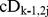 can be written by
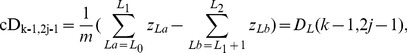
(30)

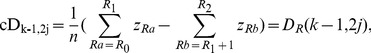
(31)where 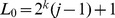, 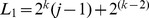, 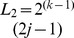; 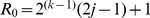, 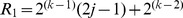, 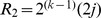, and 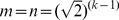. In terms of equation (30), and (31), 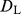 , and 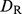 can reflect data fluctuation of two segments, namely Z_L_ and Z_R_ in Z covered by 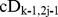 and 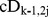, respectively. None loses of generalization, if 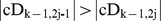 is true, it means that bigger data fluctuation exists in Z._L_ than in Z._R_. That is, a potential abrupt change exists in Z_L_ covered by 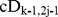 with more probability than in Z_R_ covered by 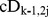. Therefore, in terms of criterion 2, left-child node 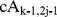 is selected to be involved into current search path in TcA, and vice versa.

In terms of two search criteria above, two HWKS-based algorithms are implemented to detect abrupt change from a diagnosed time series Z. In Algorithm 2, the distribution distance between *X* and Z is calculated in terms of a selected non-leaf node 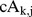 in TcA. In this function, the confidence interval is set by 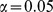, and 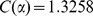. For simplicity, we only output the nodes that the value of 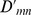 overtakes 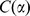, otherwise output zero. In Algorithm 3, an optimal path is detected from root to leaf nodes in TcA in terms of two search criteria, and then an estimated CP is obtained from a diagnosed Z. The pseudocodes can be found in Algorithm 2 and 3 in detail.

**Table:** 

	
**Algorithm.2.** **Input:** a normal time series input *X*, and a diagnosed time series *Z*, as well as current non-leaf node cA_k,j_ selected in TcA.**Output:** RS, the distribution distance between *X* and *Z* at the selected non-leaf node cA_k,j_ in TcA.Set *Ca* = 1.3258;RS = 0;Calculate *z*_k,j_ in terms of cA_k,j_;Sx = Calculate the e.c.d.f of z_k,j_ in X;Sz_1_ = Calculate the e.c.d.f of z^-^_k,j_ in Z;Sz_2_ = Calculate the e.c.d.f of z_k,j_ in Z;D1 = abs(Sz_1_-Sx); D2 = abs(Sz_2_-Sx);If (D1>D2) &&(D1>*Ca*) Then{Select D1; RS = D1;}elseif (D1<D2) &&(D2>*Ca*) Then{Select D2; RS = D2;}elseif (D1 = = D2) || (max (D1, D2)<*Ca*) Then RS = 0;EndifOutput RS(cA_k,j_);	**Algorithm.3.** **Input:** *X*, *Z*, TcA, and TcD derived from *Z*.**Output:** The estimated abrupt CP from TcA and TcD.Set *b* = 1; *N* = length (z); *k* = log_2_(*N*);Set the first node of optimal search path is the root node in TcA:For *i* = 1 to *k* do*a* = *k*-*i*+1;Call Algorithm 2 to calculate the distribution distance between *X* and *Z* at two non-leaf node cA_a-1,2b-1_ and cA_a-1,2b-1_ , respectively;Set S1 = RS(cA_a-1,2b-1_), S2 = RS(cA_a-1,2b_);If (S1>S2) Then {The current selected node = cA_a-1,2b-1_; *b* = 2*b*-1;} elseIf ((S1<S2) Then {The current selected node = cA_a-1,2b_; *b* = 2*b*;} elseIf ((S1 = = S2) Then {If(cD_a-1,2b-1_> cD_a-1,2b_) Then { The current selected node = cA_a-1,2b-1_; *b* = 2*b*-1; } endif If(cD_a-1,2b-1_< cD_a-1,2b_) Then { The current selected node = cA_a-1,2b_; *b* = 2*b*; } endif }} EndifEnd forOutput *b*, and *z_b_*

### D. Evaluation of HWKS

Many methods have been proposed for CP detection. In this part, the following typical methods are used to evaluate and verify the performance of the proposed HWKS method.

#### KS statistic [15]

In KS method, firstly, we divide a diagnosed sample data Z into two segments, namely, Z_m_ = {z_1_, z_2_,*…*, z_m_}, and z_N__-m_ = {z_m+1_, z_m+2_,*…*, z_N__-m_}. Then, KS statistic for these two segments is defined by,
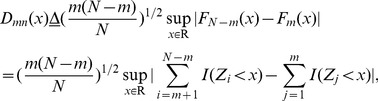
(32)where *N* is the size of the diagnosed sample Z; *m* is the size of segments *Z*_m_, that is the current diagnosed position in Z.

#### HW [28], [33]

In this method, the fluctuation coefficient vector 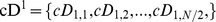 is calculated by one-level HW from a diagnosed time series Z, and then an estimated CP can be found by comparing the values of elements in vector cD^1^. The elements in cD^1^ is defined as,
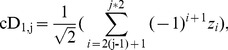
(33)where 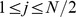. To find an abrupt change, a critical value 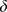 is given in terms of an identical data distribution. If 
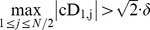
 holds true, then an abrupt CP occurs in a diagnosed sample Z.

#### T-statistic[36]

T, also known as Welch'st-test, is used only when two population variances are assumed different (the two sample sizes may or may not be equal) and hence must be estimated separately. A diagnosed sample Z is divided into Z_m_ = {z_1_, z_2_,*…*, z_m_} and Z_N__-*m*_ = {z*_m_*_+1_, z_m__+2_,*…*, z_N__-*m*_}. Then, T statistic is calculated as,
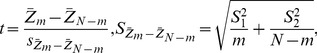
(34)where 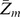, and 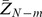 are the sample mean of two segments in diagnosed Z, S_*_ is unbiased estimator of standard deviation, and *m*, *n* is the size of two segments in Z.

## Results and Discussion

First, we evaluate the performance of HWKS on the simulated time series datasets, the sensitivity, efficiency, and accuracy of HWKS is analyzed by comparing KS, HW, and T methods. Then, we apply HWKS, and other three methods, to distinguish normal and abnormal ECG segments from the assembled ECG time series samples, and diagnose the different states of health from a patient's abnormal ECG time series segments.

### A. Analysis on simulated time series

In our simulations, the artificial time series are generated randomly in terms of normal distribution *N*(0,1), i.e., *N*(mean, *u* = 0, standard deviation, *sd* = 1), and then the normally distributed datasets is used for abrupt change detection. Specifically, each diagnosed time series sample of size *N* is composed of both a normal segment of size *k*, and an abnormal segment of size *N*-*k*, in which a designed abrupt change is contained by adding constant variation *v* to the normal random numbers of size *N*-*k*.

#### Single simulation on single CP test position

**Figure 6:**
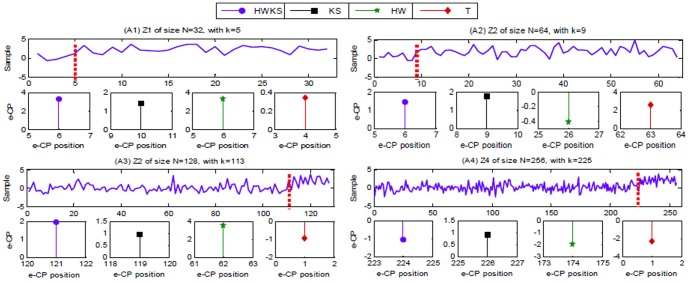
The results of single simulation on single CP test position, with constant variance *v* = 2, different sample size *N*, and CP test position *k*, by HWKS, KS, HW, and T, respectively. (A1) The estimated CP from diagnosed sample *Z*1, with *N* = 32, *k* = 5. (A2) The estimated CP from diagnosed sample *Z*2, with *N* = 64, *k* = 9. (A3) The estimated CP from diagnosed sample *Z*3, with *N* = 128, *k* = 113. (A4) The estimated CP from diagnosed sample *Z*4, with *N* = 256, *k* = 225.

First, CP detection is performed on single time series sample with single CP test position. The simulation results of CP detection are illustrated in Fig. 6, and the summarized analysis of error, and accuracy is shown in Table 1. For the proposed HWKS, it can detect the designed CP from samples of different sizes and CP test positions, with smaller error, and higher accuracy than HW, and T methods. For KS, it has the smallest averaged error in all four methods, but it has lower accuracy than HWKS, especially when sample size *N* is small. On the contrary, both of HW and T are worse, due to bigger error and lower accuracy than HWKS and KS. These simulation results indicate that HWKS has the best sensitivity and performance in four methods, especially HWKS is better than KS, due to smaller error and higher accuracy when abrupt change is located near the left or right boundary of samples with smaller size.

**Table 1:** The summarized results of single simulation with single CP test position.

*Z*M	Size, *N*CP, *k*	2^3^2	2^4^3	2^5^5	2^6^9	2^7^113	2^8^225	2^9^449	2^10^897	Averaged
**HWKS**	***Err***	***0***	***0***	***1***	***−3***	***8***	***−1***	***17***	***−10***	***5***
	***Acc***	***1.0***	***1.0***	**.** ***97***	**.** ***95***	**.** ***94***	**.** ***99***	**.** ***97***	**.** ***99***	**.** ***97***
**KS**	Err	−1	−2	5	0	6	1	−4	−9	3.5
	Acc	.88	.88	.84	1.0	.95	.99	.99	.99	.94
**HW**	Err	0	1	1	17	−51	−51	−93	−503	89
	Acc	1.0	.94	.97	.73	.60	.80	.82	.51	.79
**T**	Err	−1	−2	−1	54	−112	−224	62	126	72
	Acc	.88	.88	.97	.16	.13	.13	.88	.88	.61

#### Multiple simulations on single CP test position

**Figure 7:**
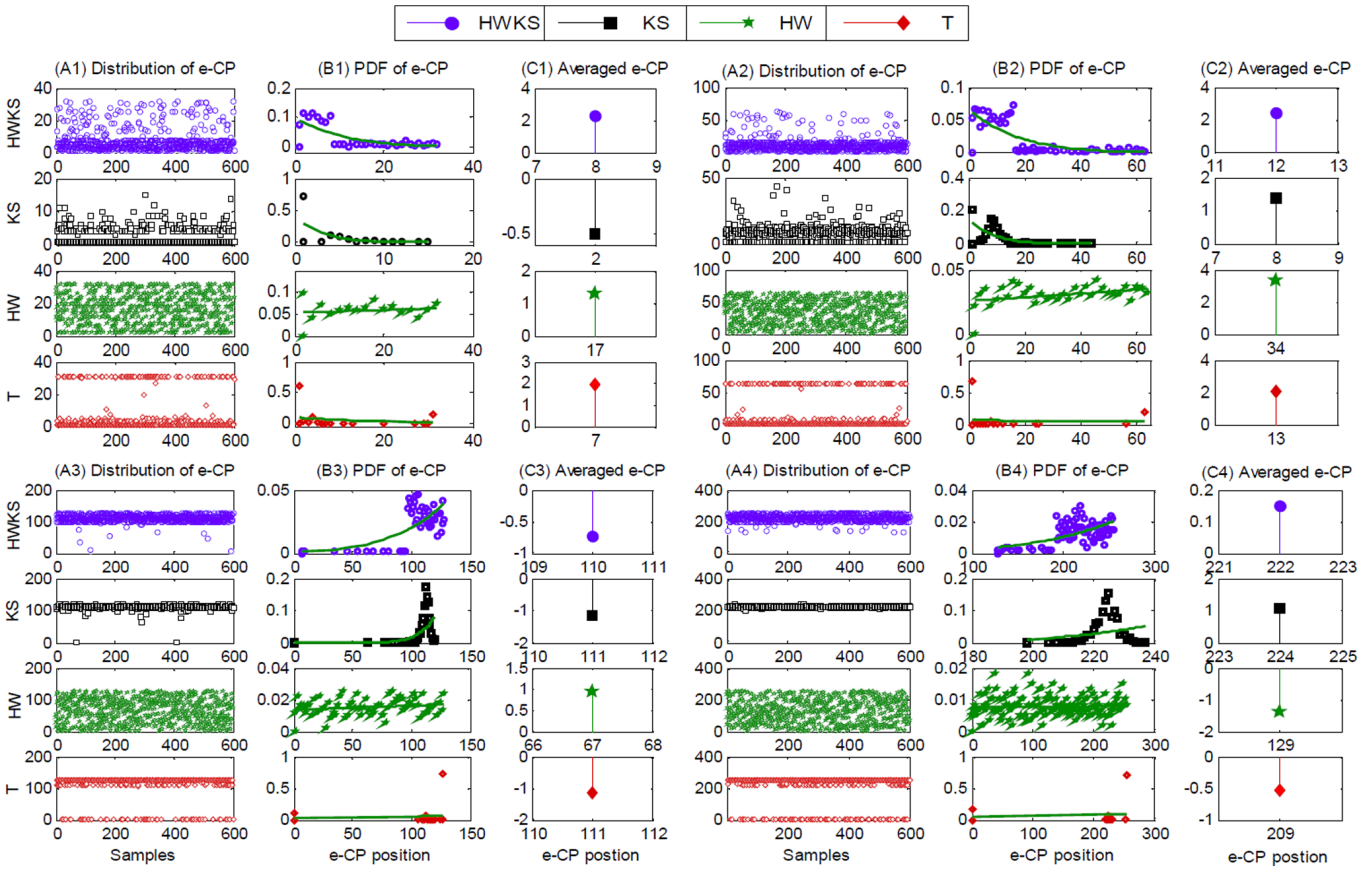
The results of multiple 600 simulations on single CP test position, with *v* = 2, different *N*, and *k*, by HWKS, KS, HW, and T, respectively. For samples with *N* = 32, *k* = 5; *N* = 64, *k* = 9; *N* = 128, *k* = 113; and *N* = 256, *k* = 225, (A1)–(A4) the distribution of e-CP, (B1)–(B4) the PDF of e-CP, and (C1)–(C4) the averaged e-CP, by HWKS, KS, HW, and T, respectively.

Second, we test HWKS and other three methods by multiple 600 simulations on single CP test position, with different *N*, and *k*. The representative results of CP detection are illustrated in Fig. 7, and the analysis of computation time, hit rate, error, and accuracy is summarized in Table 2. For the proposed HWKS, most of e-CPs are located near the designed CP position, with the shortest computation time, and the highest hit rate in all four methods, as well as smaller error, and higher accuracy than HW and T. For KS, to some extent, it is better than HWKS and HW for higher accuracy and smaller error; however, it needs much more computation time, and has lower hit rate than HWKS when abrupt change occurs near the left or right boundaries of samples with smaller size *N*. As for HW, it needs more computation time than HWKS and KS, and has the lowest hit rate and the biggest error in four methods. T is a method with the longest computation time in all four methods, and lower hit rate than HWKS and KS, although it has relatively higher accuracy than HWKS. These simulations show that, the proposed HWKS is a fast and efficient method, due to the shortest computation time and the highest hit rate in all four methods, as well as smaller error and higher accuracy than HW and T. In addition, HWKS has better sensitivity to less significant data fluctuation in sample with small size *N*, than KS, HW, and T, especially when CP is located near the left or right boundary.

**Table 2:** The summarized results of multiple 600 simulation on single CP test position.

*Z*M	Size, *N*CP, *k*	2^3^2	2^4^3	2^5^5	2^6^9	2^7^113	2^8^225	2^9^449	2^10^897	Averaged
**HWKS**	***Tim***	.*17*	.*23*	.*27*	.*33*	.*41*	.*47*	.*60*	.*75*	.*40*
	***Hit***	.*39*	.*15*	.*10*	.*06*	.*03*	.*03*	.*01*	.*01*	.*10*
	***Err***	*2*	*2*	*3*	*3*	*−3*	*−3*	*−5*	*3*	*3*
	***Acc***	.*75*	.*88*	.*91*	.*95*	.*98*	.*99*	.*99*	.*99*	.*93*
**KS**	Tim	.09	.15	.36	.68	1.6	3.4	9.05	23.8	4.9
	Hit	.0	0	.08	.14	.14	.13	.10	.09	.09
	Err	−1	−2	−3	−1	−2	−1	−1	1	1.5
	Acc	.88	.88	.91	.98	.98	.99	.99	.99	.95
**HW**	Tim	.05	.06	.26	.70	1.15	4.1	8.7	29.7	5.6
	Hit	0	0	0	0	0	0	0	0	0
	Err	1	4	12	25	−46	−95	−186	−380	93.6
	Acc	.88	.75	.63	.61	.64	.63	.64	.63	.67
**T**	Tim	.6	1.1	2.2	4.5	9.2	17.7	36.7	75.2	18.4
	Hit	.02	.017	.02	.01	.015	.01	.01	.01	.014
	Err	0	1	2	4	−2	−16	−27	−46	12.3
	Acc	1.0	.94	.94	.94	.98	.94	.95	.96	.95

#### Multiple simulations on different CP test positions

**Figure 8:**
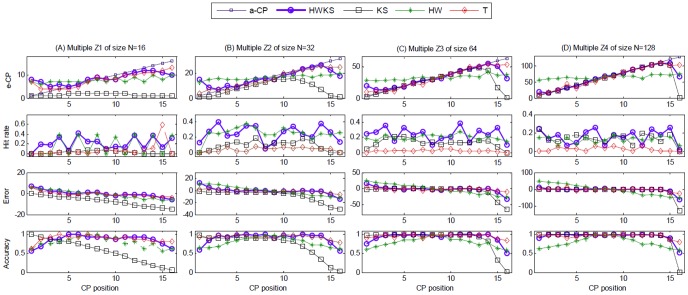
The analysis of e-CP, hit rate, error and accuracy for multiple 100 simulations on different CP test positions, with different *N*, and *k*, by HWKS, KS, HW, and T, respectively. (A) The results of multiple samples *Z*1, with *N* = 16; (B) the results of multiple samples *Z*2, with *N* = 32; (C) the results of multiple samples *Z*3, with *N* = 64; (D) the results of multiple samples *Z*4, with *N* = 128.

**Figure 9:**
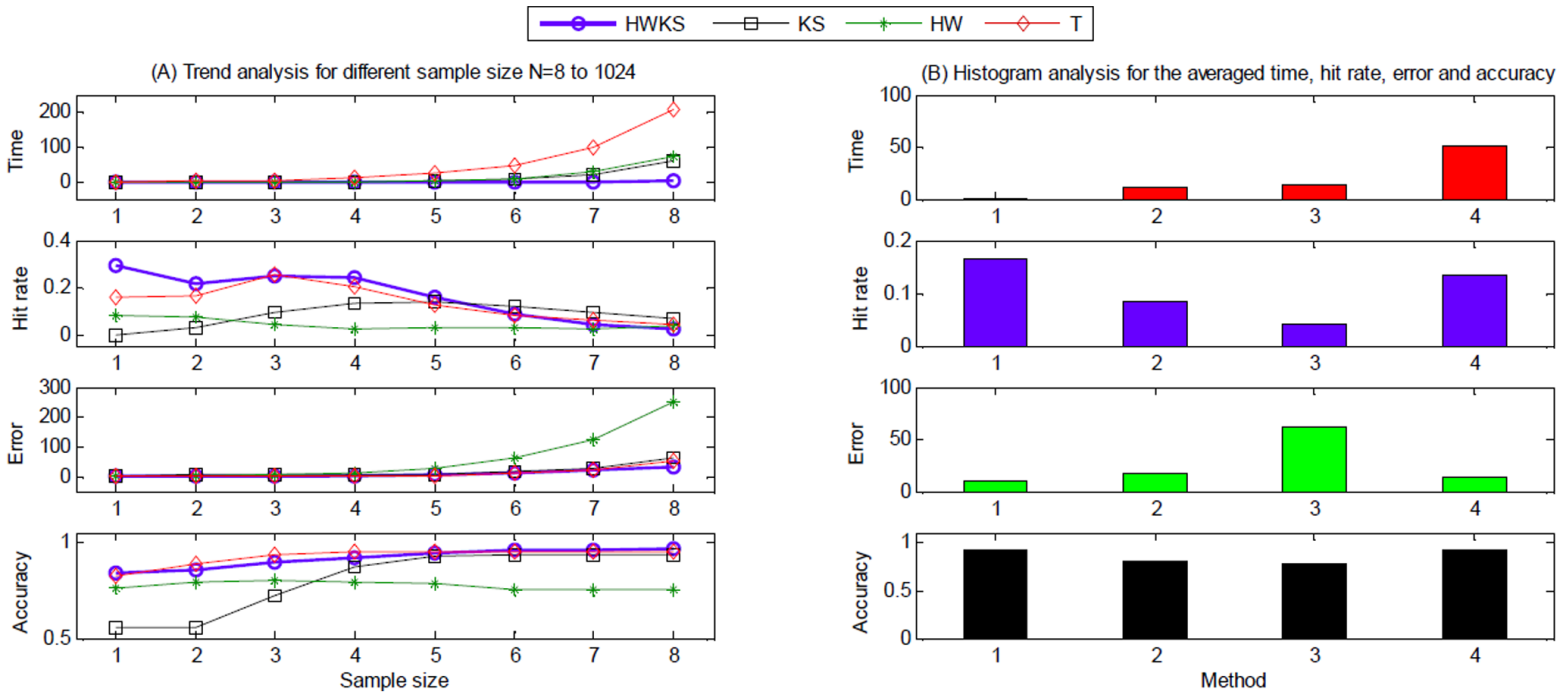
The analysis of computation time, hit rate, error and accuracy on different sample size, for HWKS, KS, HW, and T, respectively. (A) The trend analysis for different sample size from *N* = 2^3^ to 2^10^, and (B) the histogram analysis for the averaged computation time, hit rate, error and accuracy. In (B), ‘1’ stands for HWKS, ‘2’ stands for KS, ‘3’ stands for HW, and ‘4’ stands for T.

Third, for each diagnosed sample group, multiple 100 simulations on different CP test positions are performed by the proposed HWKS and other three methods. In our simulations, for each *N*, we select different 16 CP test positions from the different parts of diagnosed samples, *i*.*e*., the CP test position is designed by 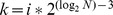, 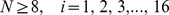. The selected results of simulations on single CP with different test positions are shown in Fig. 8, including e-CP, hit rate, error, and accuracy. In addition, the simulation results for different samples of size from 2^3^ to 2^10^ are summarized in Table 3. In terms of computation time, hit rate, error, and accuracy, the trend analysis for different sample size *N*, as well as the histogram of the averaged analysis for different methods are plotted in Fig. 9.

**Table 3:** The summary of multiple simulations on different CP test positions.

M*Z*	Size, *N*	2^3^	2^4^	2^5^	2^6^	2^7^	2^8^	2^9^	2^10^	Averaged
**HWKS**	***Tim***	.*18*	.*45*	.*59*	.*78*	.*86*	*1.18*	*1.71*	*2.6*	*1.04*
	***Hit***	.*29*	.*21*	.*25*	.*24*	.*16*	.*10*	.*05*	.*03*	.*17*
	***Err***	*1.25*	*2.2*	*3.2*	*4.9*	*6.8*	*11.2*	*21.6*	*33.7*	*10.6*
	***Acc***	.*84*	.*86*	.*90*	.*92*	.*95*	.*96*	.*96*	.*97*	.*92*
**KS**	Tim	.14	.50	.91	1.8	4.1	9.3	22.9	62.7	12.79
	Hit	.0	.03	.09	.13	.14	.11	.09	.07	.08
	Err	3.5	7.1	8.8	8.1	9.5	16.1	31.0	63.3	18.4
	Acc	.56	.56	.72	.87	.92	.93	.94	.94	.81
**HW**	Tim	.03	.18	.45	.77	2.2	8.2	28.7	72.7	14.15
	Hit	.08	.08	.04	.02	.02	.03	.02	.03	.04
	Err	1.9	3.3	6.4	13.2	27.3	61.8	123.7	251.9	61.2
	Acc	.76	.79	.79	.79	.78	.76	.76	.75	.77
**T**	Tim	.78	3.1	6.1	12.1	24.3	49.7	101.7	209.6	50.9
	Hit	.15	.16	.25	.20	.13	.08	.06	.04	.13
	Err	1.38	1.8	2.1	3.1	5.7	12.4	25.3	53.3	13.1
	Acc	.83	.88	.93	.95	.95	.95	.95	.94	.92

For the proposed HWKS, it has the best performance for CP detection from samples Z of size *N* from 2^3^ to 2^7^, because of the shortest computation time, the highest hit rate, the smallest error, and the highest accuracy in all four methods. Meanwhile, HWKS is better than KS, HW, and T methods for samples Z of size *N* from 2^8^ to 2^10^, due to the shortest computation time, the smallest error, and the highest accuracy in all four methods, except that hit rate is slightly lower than KS and T. For KS, in general, it has shorter computation time, bigger hit rate, smaller error and higher accuracy than HW. However, KS has the lowest hit rate, the biggest error, and the lowest accuracy as *N* is below 2^5^ in all four methods, which means that KS has worse sensitivity and performance for less significant data fluctuation, especially when *N* is smaller. For HW, it generally takes shorter computation time than T, whereas, it has the smallest hit rate, the biggest error and lowest accuracy in all four methods. For T, it takes the longest computation time, although bigger hit rate, smaller error and higher accuracy than KS and T. These results show that HWKS has better performance and sensitivity to less significant data fluctuation near the left and right boundary of samples with smaller size *N* and HWKS is an encouraging method for CP detection on simulated time series, due to shorter computation time, higher hit rate, smaller error, and higher accuracy than KS, HW, and T methods.

### B. Analysis on ECG time series

To verify the performance of the proposed method further, we apply HWKS, and KS, HW, and T methods, to detect abrupt change from ECG time series provided by PhysioBank. In ECG experiments, we design the diagnosed ECG samples from different ECG datasets, including the MIT-BIH Normal Sinus Rhythm Database (NSRDB) [37], MIT-BIH Noise Stress Test Database (NSTDB) [38], and MIT-BIH Malignant Ventricular Arrhythmia Database (MVADB) [39], [40].

#### CP detection from assembled ECG samples

First, we select a normal ECG dataset, *16265m* from the NSRDB, and an abnormal ECG dataset, *118e00m* from the NSTDB, and then assemble the diagnosed ECG samples from different segments in the *16265m* and *118e00m*. Specifically, we take the normal ECG segment of size *m* as *X*_m_, and the abnormal segment of size *n* as *Y*_n_, respectively, and then assemble the diagnosed ECG sample Z = {*X*_m_ , *Y*_n_} = {x_1_,…, x_m_, y_1_,…, y_n_}. Meanwhile, we design another normal ECG segment from *16265m*, i.e., *X* = {x_1_,…, x_m+n_}, as normal time series input.

**Figure 10:**
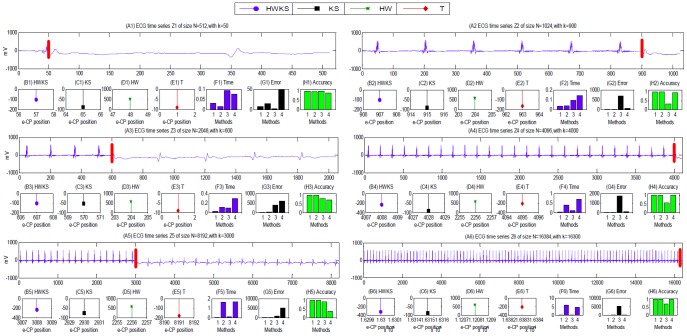
The results of CP detection from assembled ECG time series of size *N* = 2*^k^*, *k* = 9, 10, …, 14, by HWKS, KS, HW, and T, respectively. (A1)–(A6) the assembled ECG sample *Z*1–*Z*6; (B1)–(B6),(C1)–(C6) ,(D1)–(D6) ,(E1)–(E6) the e-CP detected from *Z*1–*Z*6, by HWKS, KS, HW, and T, respectively; (F1)–(F6) the diagram analysis for the computation time, (G1)–(G6) the error of e-CP, and (H1)–(H6) the accuracy for *Z*1–*Z*6, respectively. In (F)-(H), ‘1’ stands for HWKS, ‘2’ stands for KS, ‘3’ stands for HW, and ‘4’ stands for T.

In this ECG experiment, a single CP test position is arranged near the left and right boundary of the assembled ECG sample. For the assembled ECG sample of size from *N* = 2^9^ to 2^14^ with different CP position *k*, the results of CP detection from Z1–Z6 are illustrated in Fig. 10, and then the analysis of computation time, error, and accuracy are summarized in Table 4. Comparing with KS, HW, and T methods, the results show that the proposed HWKS can estimate CP position more quickly, and distinguish the normal and abnormal segments from the assembled ECG samples more efficiently, with smaller error and higher accuracy. For KS, it has smaller error and higher accuracy than HW and T, whereas, it takes much more computation time than HWKS, HW, and T, especially when sample size *N* gets bigger, meanwhile, KS is less sensitive for less significant statistic fluctuation, with bigger error near the right boundary. For HW, it is inefficient, because of the biggest error and lowest accuracy in all four methods. For T, it is also discouraging for longer computation time, bigger error and lower accuracy than HWKS and KS. Therefore, the proposed HWKS has the best performance in this ECG experiment out of all four methods.

**Table 4:** The summary of CP detection from the assembled ECG samples.

*Z*M	Size, *N*CP, *k*	2^9^50	2^9^500	2^10^300	2^10^900	2^11^600	2^11^1400	2^12^1600	2^12^4000	2^13^3000	2^13^8100	2^14^5000	2^14^16000	Averaged
**Time**	*HWKS*	.*032*	.*036*	.*032*	.*033*	.*035*	.*034*	.*034*	.*034*	.*041*	.*036*	.*060*	.*049*	.*038*
	KS	.014	.017	.037	.038	.116	.118	.395	.397	1.59	1.46	5.23	5.58	1.25
	HW	.092	.089	.093	.095	.094	.080	.081	.091	.091	.087	.085	.092	.089
	T	.074	.079	.149	.141	.296	.293	.718	.696	1.65	1.66	4.39	4.38	1.21
**Error**	*HWKS*	*7*	*0*	*0*	*7*	*7*	*21*	*0*	*8*	*8*	*2*	*12*	*0*	*6*
	KS	15	227	18	15	30	28	18	28	70	6	3	15	39.4
	HW	2	296	96	696	396	1196	1396	1744	744	3762	662	5092	1340.1
	T	49	11	299	63	599	647	2495	95	5191	91	11383	83	1750.5
**Accuracy**	*HWKS*	.*98*	*1.0*	*1.0*	.*99*	.*99*	.*99*	*1.0*	.*99*	.*99*	.*99*	.*99*	*1.0*	.*99*
	KS	.97	.55	.98	.98	.98	.98	.99	.99	.99	.99	.99	.99	.95
	HW	.99	.42	.90	.32	.80	.41	.66	.57	.90	.54	.95	.68	.68
	T	.90	.97	.70	.93	.70	.68	.39	.97	.36	.98	.30	.99	.74

#### CP detection from abnormal ECG samples

**Figure 11:**
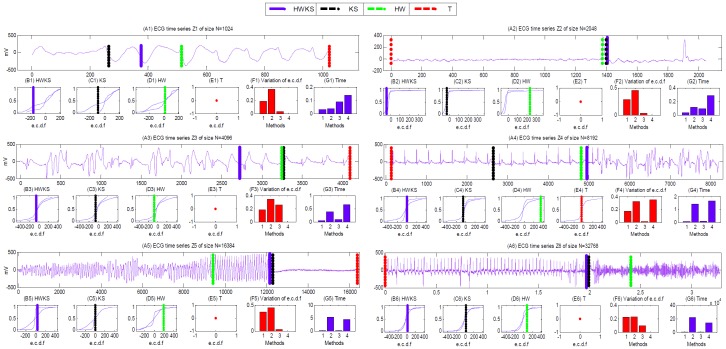
The results of CP detection from abnormal ECG time series of size *N* = 2*^k^*, *k* = 10, 11, …, 15, by HWKS, KS, HW, and T, respectively. (A1)–(A6) the abnormal ECG sample *Z*1–*Z*6; (B1)–(B6), (C1)–(C6), (D1)–(D6), (E1)–(E6) the e.c.d.f derived from two segments of *Z*1–*Z*6, by HWKS, KS, HW, and T, respectively; (F1)–(F6) the diagram analysis of the distance of e.c.d.f, and (G1)–(G6) the computation time of HWKS, KS, HW, and T in *Z*1–*Z*6, respectively. In (F)–(G), ‘1’ stands for HWKS, ‘2’ stands for KS, ‘3’ stands for HW, and ‘4’ stands for T.

To verify the performance of CP detection further, we apply the proposed HWKS, and KS, HW and T to analyze the abnormal ECG time series directly. In this part, we select the abnormal ECG segment from *118e00m* in the NSTDB, i.e., Z = {y_1_,…, y_n_}, as a diagnosed ECG sample. Then, we take another normal ECG segment from *16265m* in the NSRDB, i.e., Z = {*X*_n_ } = {x_1_,…, x_n_ }, as normal input signal. To some extent, the distance of e.c.d.f can partly reflect the statistical fluctuation. Therefore, we take this variable as an indicator of the data fluctuation between two ECG segments divided by e-CP position. The results of CP detection, including the e-CP position, distance of e.c.d.f, and computation time, are plotted in Fig. 11, and summarized in Table 5.

**Table 5:** The summary of CP detection from abnormal ECG samples.

M*Z*	Size, *N*	2^10^	2^11^	2^12^	2^13^	2^14^	2^15^	Averaged
**e-CP**	*HWKS*	*376*	*1405*	*2722*	*4945*	*12150*	*19711*	*NA*
	KS	264	1399	3268	2646	12296	19930	NA
	HW	514	1374	3244	4810	9416	24066	NA
	T	1023	1	4095	132	16383	1	NA
**Computation time**	*HWKS*	.*032*	.*034*	.*034*	.*037*	.*049*	.*075*	.*043*
	KS	.036	.114	.376	1.41	5.19	20.82	4.66
	HW	.090	.093	.092	.095	.096	.115	.097
	T	.138	.286	.657	1.65	4.38	13.79	3.49
**Variance of e.c.d.f**	*HWKS*	.*183*	.*287*	.*183*	.*168*	.*359*	.*223*	.*234*
	KS	.362	.454	.345	.326	.442	.229	.358
	HW	.029	.025	.254	.008	.030	.096	.073
	T	0	0	0	.347	0	0	.057

For abnormal ECG samples Z1–Z6 with different size *N* from 2^10^ to 2^15^, HWKS can detect abrupt change position, and then divide the original ECG sample into two parts, with the shortest computation time out of four methods, and bigger distance of e.c.d.f than HW and T methods. For KS, it can detect CP with the maximal distance of e.c.d.f; however, it needs longer computation time than HWKS, HW and T, especially for ECG sample with big size *N*. On the other hand, for HW, it is inefficient, due to smaller distance of e.c.d.f than HWKS and KS. T is also inefficient, because of longer computation time than HWKS, HW, the smallest distance of e.c.d.f, and the invalid e-CP position for most of the abnormal ECG samples.

Especially, the results of CP detection from Z1–Z3 plausibly indicate that, a patient seems recovering from abnormal state of health, after overtaking the critical e-CP position detected by HWKS. On the contrary, the results from Z4–Z6 suggest that, a patient is encountering a risky situation from the former state of health, after going through the vital e-CP position. These results indicate that HWKS can capture abrupt change position from a diagnosed ECG sample quickly and efficiently, and the detected CP is very useful to find a critical time from ECG time series, where a patient might encounter an important conversion between two different states of health. Therefore, HWKS is an efficient and encouraging method for detecting abrupt change from abnormal ECG time series, and it is very meaningful in inspecting and diagnosing different states of health from diagnosed ECG time series more quickly and efficiently.

## Conclusion

In this paper, based on HW and a modified KS statistic, a novel HWKS method is proposed for CP detection from large-scale time series. First, two BSTs are constructed from a diagnosed time series by means of multi-level HW method, the framework of HWKS method is implemented by introducing a revised KS statistic and two search criteria based on TcA and TcD; and then two HWKS-based algorithms are designed to detect an optimal path from TcA in terms of two search criteria. Second, the performance of HWKS is analyzed on simulated time series; the simulations show that HWKS is more sensitive and efficient than KS, HW, and T methods, especially when CP occurs near the left or right boundary with less significant data fluctuation in time series of small size. Last, HWKS is applied to analyze abrupt change on both assembled and abnormal ECG datasets. The results indicate that HWKS can successfully detect abrupt change, and distinguish normal and abnormal ECG segments from assembled ECG samples. In addition, HWKS can estimate an abrupt CP from abnormal ECG segments with different time-scale, and then divide it into two adjacent parts with maximal data fluctuation; therefore, it is very useful to diagnose a patient's different states of health from an abnormal ECG segment more quickly and efficiently. In conclusion, HWKS is a novel and efficient method for fast CP detection; it is a very powerful and promising tool to find useful information from large-scale time series databases.
